# An In Vitro Examination of Whether Kratom Extracts Enhance the Cytotoxicity of Low-Dose Doxorubicin against A549 Human Lung Cancer Cells

**DOI:** 10.3390/molecules29061404

**Published:** 2024-03-21

**Authors:** Asep Bayu, Siti Irma Rahmawati, Firmansyah Karim, Jonathan Ardhianto Panggabean, Dasilva Primarindu Nuswantari, Dwi Wahyu Indriani, Peni Ahmadi, Rendi Witular, Ni Luh Putu Indi Dharmayanti, Masteria Yunovilsa Putra

**Affiliations:** 1 Research Center for Vaccine and Drugs, National Research and Innovation Agency (BRIN), Jalan Raya Jakarta-Bogor KM 46, Cibinong, Bogor 16911, Indonesia; siti.irma.rahmawati@brin.go.id (S.I.R.); firmansyahkarim93@gmail.com (F.K.); jonathanardhianto@gmail.com (J.A.P.); dasilvarindu@gmail.com (D.P.N.); dwiw007@brin.go.id (D.W.I.); peni.ahmadi@brin.go.id (P.A.); 2 Ministry of Health, Republic of Indonesia, Jalan HR Rasuna Said Blok X5 Kavling 4-9, Kuningan, South Jakarta 12950, Indonesia; rendyaw@yahoo.com; 3 Research Organization for Health, National Research and Innovation Agency (BRIN), Jalan Raya Jakarta-Bogor KM. 46, Cibinong, Bogor 16911, Indonesia; nilu011@brin.go.id

**Keywords:** kratom, *Mitragyna speciosa*, alkaloid, mitragynine, cytotoxicity, anticancer

## Abstract

Doxorubicin is an effective chemotherapeutic agent in the treatment of solid hematological and non-hematological carcinoma. However, its long-term usage could result in side effects, such as cardiomyopathy, chronic heart failure, neurotoxicity and cancer cell resistance. In this study, we reported the sensitivity enhancement of A549 human lung cancer cells on doxorubicin at a low dose (0.1 ppm) in combination with 10–60 ppm of crude and alkaloid extracts derived from the leaves of Kratom (*Mitragyna speciosa* (Korth.) Havil. Rubiaceae). A549 cancer cell lines were insensitive to the crude extract containing low mitragynine (MG) (4–5%), while these cells were moderately inhibited by the alkaloid extract containing 40–45% MG (IC_50_ of 48–55 ppm). The alkaloid extract was found to inhibit A549 cancer cells via apoptosis as suggested by the higher relative fluorescence intensity with Annexin compared to that in propidium iodide (PI), i.e., a positive Annexin and a negative PI. The combination of crude extract and doxorubicin sensitized A549 cancer cells to doxorubicin by 1.3 to 2.4 times, while the combination with the alkaloid induced a 2.6- to 3.4-fold increase in sensitivity. The calculated combination index (CI) for doxorubicin with the crude and alkaloid extracts was 0.6 and 0.3, respectively, showing potential synergistic combinations to reduce the level of dosage of doxorubicin used in chemotherapy. In addition, the synergistic enhancement effect of crude extract on the cytotoxic activity of doxorubicin provides insights into the plausibility of non-alkaloids to influence the biological activities of Kratom.

## 1. Introduction

Doxorubicin is one of the anthracyclines that are widely used as chemotherapeutic agents in the treatment of solid and hematological malignancies [1]. Although doxorubicin is highly effective as a chemotherapeutic agent in the treatment of many solid tumors and blood and gynecological cancers, its use has been considered to be associated with both acute and chronic adverse cardiovascular events [2]. Cumulative doses frequently induce cardiomyopathy and chronic heart failure, with prevalences between 4 and 36% and 0.2 and 8.75%, respectively [3]. Chemotherapy for hepatocellular carcinoma patients with doxorubicin has also been reported to lead to side effects, such as septicemia, neuropathy, nephrotoxicity and cardiotoxicity [4]. Furthermore, doxorubicin usage exhibits neurotoxicity that could cause severe brain damage, anxiety, depression and poor cognitive performance [5]. As such, the use of doxorubicin could also affect the quality of life and long-term survivorship of cancer patients. Thus, novel therapeutic approaches are being developed to minimize the side effects associated with the administration of doxorubicin.

Combining chemotherapeutic drugs is a promising strategy that can improve treatment efficacy, avoid drug resistance and reduce treatment duration, even at concentrations lower than those used for monotherapy [4,6]. This strategy was first introduced by Freireich and co-workers, who successfully treated pediatric patients with acute lymphocytic leukemia by combining methotrexate, 6-mercaptopurine, vincristine and prednisone [7]. In another study, a combination of sabutoclax and minocycline displayed selective toxicity and a reduction in tumor growth in vitro and was safely efficacious in vivo when used to treat pancreatic ductal adenocarcinoma [8]. The work exhibits the plausibility of combination therapy to minimize the toxic effects on normal cells, while it is simultaneously cytotoxic on cancer cells. In fact, conventional monotherapeutics face two main issues. First, techniques based on conventional monotherapeutics non-selectively target actively proliferating cells, which destroys normal as well as cancerous cells. As a result, the treatment could have multiple side effects and present risks to the body. Secondly, therapy using a single compound could induce cancer cells to recruit alternative salvage pathways; as such, the cancer cells would adapt to the exposure to the compound, resulting in drug resistance [7]. In fact, about 90% of cases of cancer therapy failure and 80–90% of cancer deaths are related to cancer resistance [3].

In some clinical trials, combinations of chemotherapeutic drugs and natural products have been postulated to possess similar effects to conventional chemotherapeutic drugs but with fewer adverse effects [3]. In fact, natural products and their analogs have provided approximately 30–34% of Food and Drug Administration-approved drugs [9,10]. Among them, alkaloids attract much interest due to their multiple biological activities that are advantageous for drug discovery and development. Several alkaloids have been evaluated for their chemotherapeutic adjuvant effect with doxorubicin against several human cancer cell lines like prostate, lung, cervix and colorectal [3]. For instance, neferine isolated from the seed embryo of lotus (*Nelumbo nucifera*) sensitized A549 human lung cancer cells to low doses of doxorubicin and affected cardiomyocyte (H9C9) cell viability toward doxorubicin [11].

Recently, much attention has been given to alkaloids in the leaves of Kratom (*Mitragyna speciosa* (Korth.) Havil. Rubiaceae) because Kratom’s alkaloids offer a multitude of potential medicinal applications [12]. In particular, their attractiveness is related to their agonistic action on the human μ-opioid receptor (hMOR), which can be used as an opioid alternative [9,12,13,14], analgesic [10,15] and antidepressant [16]. Herein, mitragynine (MG) and its oxidized analogs, viz., 7-hydroxymitragynine (7-OHMG), are commonly the main targeted Kratom alkaloids because they show multiple responses to hMOR agonists, i.e., EC_50_ = 339 nM, E_max_ = 34% and EC_50_ = 34.5 nM, and E_max_ = 47%, respectively, and competitive antagonists at the κ- as well as δ-opioid receptors (KOR, DOR) and G-protein-biased agonists of MOR [17]. Other minor alkaloids, such as paynantheine (PAY) and speciogynine (SPG), also show activity to inhibit twitch contraction, while corynantheidine exhibits MOR antagonism [18]. 

Despite the increasing number of studies on Kratom’s alkaloids as opiate withdrawal substitutes, current research on the cytotoxicology of Kratom’s alkaloids is increasing because plant-based indole alkaloids are mostly active against cancer cells [19]. MG is the main indole alkaloid derived from Kratom’s leaves. It was found to be potent in an in vitro study against K562 erythroid leukemic cell lines (IC_50_ = 25 μM) and HCT 116 colon carcinoma (IC_50_ = 47 μM) after 48 h exposure [20]. Its alkaloid derivatives like SPG, PAY and speciociliatine (SPC) were cytotoxic towards nasopharyngeal carcinoma cell lines (NPC/HK1 and C666-1) with IC_50_ of >32 μM after 72 h exposure) [21]. Furthermore, MG inhibited cancer cells in the central nervous system, i.e., C6 rat glioma and SH-SY5Y human neuroblastoma, with IC_50_ values of 168 μM and 146 μM, respectively [22]. In addition to its pure alkaloids, Kratom extracts (~45% MG) obtained from the ultrasonication of numerous commercial Kratom products were reported to be cytotoxic against Caco-2 human colon cancer cells with IC_50_ values of 3–17 μg mL^−1^ (8–43 μM) after 24 h exposure [23]. Furthermore, Domnic et al. reported that the cytotoxicity of an alkaloid-enriched extract (~29% MG) against NPC/HK1 cells line was approximately five times greater than that of a methanolic crude extract (~1%) (IC_50_ = 32 μg mL^−1^ (80 μM) and 134 μg mL^−1^ (336 μM), respectively) [21]. 

Interestingly, Domnic et al. observed that the single treatment with MG, SPG, PAY and SPC against NPC/HK1 and C666-1 carcinoma cell lines did not significantly increase cytotoxicity at low doses (<10 μM) [21]. Meanwhile, their isolated alkaloid fractions contained two compounds of MG and SPC and showed more potency towards NPC/HK1 compared with the respective alkaloids solely (27–50 μM and >75 μM, respectively). While screening the cytotoxicity of the extracts derived from commercial Kratom products against Caco2 cell lines, Oliveira et al. also observed a 2- to 14-fold increase in the cytotoxicity of the extracts compared with MG [23]. These reports suggest that the in vitro cytotoxic activity of Kratom is affected by the presence of the multi-components of its alkaloids. These findings are supported by results showing that a combination of MG and SPC increased the sensitivity of nasopharyngeal carcinoma cells to cisplatin four times more at a concentration close to its IC_50_ values [21]. 

Shi et al. reported that the overexpression of Cyclooxygenase-2 (COX-2) increases nasopharyngeal carcinoma cell proliferation and is associated with the resistance of this cancer cell toward cisplatin [24]. Combining cisplatin with a COX-2 inhibitor, e.g., celecoxib, was reported to resensitize nasopharyngeal carcinoma cells to cisplatin [25]. Since MG was active in inhibiting COX2-mRNA and protein expression [26], Domnic et al. speculated that the downregulation of COX-2 by Kratom’s alkaloids contributed to sensitizing nasopharyngeal carcinoma cells to cisplatin in their combination [21]. The combination of celecoxib and doxorubicin has also been shown to improve the inhibition of A431 human skin cancer cells [24], B16 murine melanoma tumor cell lines [27], 4T1 mouse breast cancer cell lines [28], MCF-7 human breast cancer cell lines [29] and A549 human lung cancer cell lines [30]. 

Based on the results outlined above, we investigated the cytotoxicity of standard extracts of crude (MG-low) and alkaloid (MG-rich) Kratom in comparison with doxorubicin against A549 human lung cancer cells. Apoptosis’ effect on the Kratom alkaloid was confirmed by using a fluorescence microscope following the Annexin V/PI staining protocol. We observed a 1.3- to 3.4-fold sensitivity enhancement of doxorubicin at a low dosage (0.1 ppm) against A549 human lung cancer cells when used in combination with the Kratom extracts. The results indicate that Kratom could be used for combination therapy with commercial anticancer drugs, as suggested by the small calculated combination index (CI) (<1.0) for doxorubicin with both extracts. 

## 2. Results and Discussion

At first, the standardization of the Kratom extract was performed to obtain a stable and repeatable extract in terms of the extract quality resulting from the laboratory and pilot-scale productions. Cascade conventional extraction using organic solvents followed by acid–base treatments was performed because this protocol is commonly used and applicable for large-scale production. In the first step, a preliminary study was carried out to explore the effects of the use of different organic solvent types, extraction times and temperatures on the extract yield and MG production in Kratom’s crude extract. Afterward, the optimized condition was applied at the pilot scale (~100 L) in order to determine the stability of the extraction of the crude and alkaloid extracts.

### 2.1. Kratom Crude Extract Preparation

**Figure 1:**
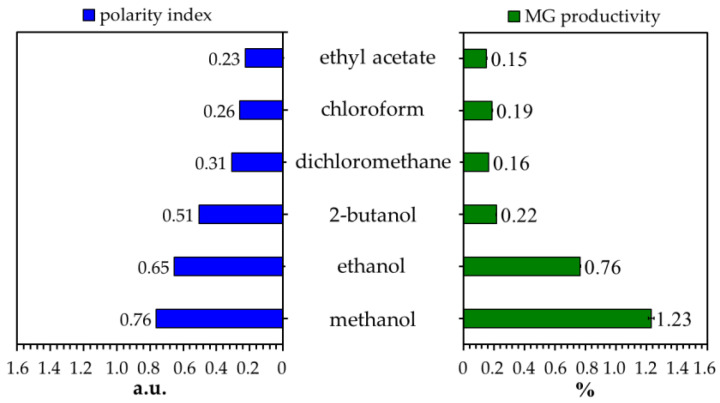
Effects of different solvent types on the MG content of Kratom’s crude extract. The polarity of each solvent is based on the literature [34].

**Figure 2:**
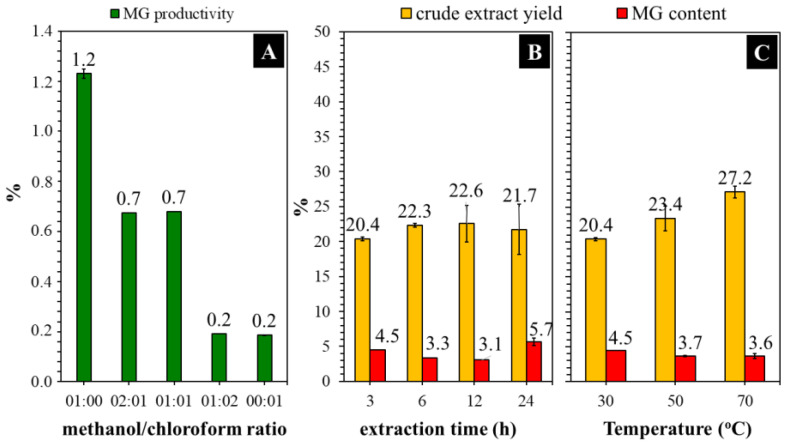
Effects of the use of methanol/chloroform ratio (**A**), the extraction time at 30 °C (**B**) and temperature at 3 h over methanolic extraction (**C**) of Kratom leaf powder.

Several organic solvents with different polarities were evaluated to determine their effectiveness in MG extraction from the Kratom leaf powder. We focused on analyzing MG because it is the main and most attractive alkaloid in Kratom’s leaves. The sample of Kratom leaves contained ~1.4% dry weight of MG (Table 1). This is aligned with many reports mentioning that the typical constituent of MG is in the range of 0.8–2.0% of the leaf mass or approximately two-thirds of the total alkaloid content [12,31,32]. As expected, the obtained MG was in line with the polarity of the solvent used (Figure 1). Herein, short-chain alcohols like methanol and ethanol exhibited the best performance in extracting MG from Kratom leaves. To ensure this, we investigated the co-solvent effects of methanol and chloroform on the extraction process because these mixtures are commonly used in biomass extraction [23,33]. Once again, extraction using a high proportion of methanol showed better MG production (Figure 2A).

As a product of plant metabolism, phytochemicals, like alkaloids, are typically stored in the internal environment of living cells [35]. The intracellular components are enclosed by the plasma membrane, which acts as a protective layer. As such, an effective destruction of the cell’s barrier is critical to maximally releasing them. Alcohols like methanol and ethanol can increase membrane permeability, resulting in increased fluid and permeability (swelling). These small and highly polar compounds can penetrate a membrane effectively by destructing the intra-/intermolecular bonds of the membrane metabolites [36], and thus, the cells start to break down and release more intracellular materials. The results indicate that crude extracts derived from alcohols contained more polar compounds, as indicated by chromatographic separation using the RP-phase column at the beginning (Figure S1, Supplementary Information). In fact, methanol [37,38] and ethanol [39] are commonly used to produce MG-rich extract from Kratom leaves. Therefore, we selected methanol for further experiments in the crude extraction step.

Figure 2B shows that the yield of crude extract increased to some extent when the extraction time was increased from 3 to 12 h. Extending the time further to 24 h did not significantly affect the yield. Nevertheless, the MG content was observed to increase by about 1.3 times compared with that obtained from a 3 h reaction. A long extraction time is generally beneficial for producing phytochemicals with a high yield. This is because when all metabolites are released inside the leaf, time is required for the solvent to contact and break the cell’s barrier. Meanwhile, the extract yield was insignificantly increased (~1.3-fold) by increasing the extraction temperature from 30 to 70 °C (Figure 2C). The MG content was observed to be around 3.7–4.5%. Considering the energy consumption and safety concerns surrounding heat-assisted extraction with insignificant changes in performance, conducting the experiment under a normal temperature seems to be more appropriate in terms of economic cost.

### 2.2. Kratom Alkaloid Extract Preparation

The alkaloid extract was derived from the acid–base treatment of Kratom’s crude extract. We selected methanol as a solvent in the crude extract preparation as a first step because its results showed good efficiency. During this extraction, the use of methanol should be strictly considered. Our conditions required the extraction to be carried out using methanol at least four times to ensure MG was completely removed from the leaves (Table S1, Supplementary Information). The maximum yield and MG content of the crude extract were about 41% and 5%, respectively (Table 1, run 1). Meanwhile, the alkaloid yield was in the range of 3% with an MG content of ~45% (Table 1, run 1). The alkaloid results are in line with previous research that used “red Indonesian micro powder” supplied by Moon Kratom (Austin, TX) [9,40,41,42]. The MG peak was confirmed to have a typical UV absorption for MG; i.e., maximum at 220–225 nm and shoulders at 245–247, 285 and 291–293 nm (Figure S2, Supplementary Information). This observation is in line with previous publications [43,44]. The HPLC peak chromatograms of the crude extract indicate the presence of other alkaloids, such as 7-OHMG, paynantheine (PAY), speciogynine (SPG) and speciociliatine (SPC), in significant amounts (Figure S3, Supplementary Information).

**Figure 3:**
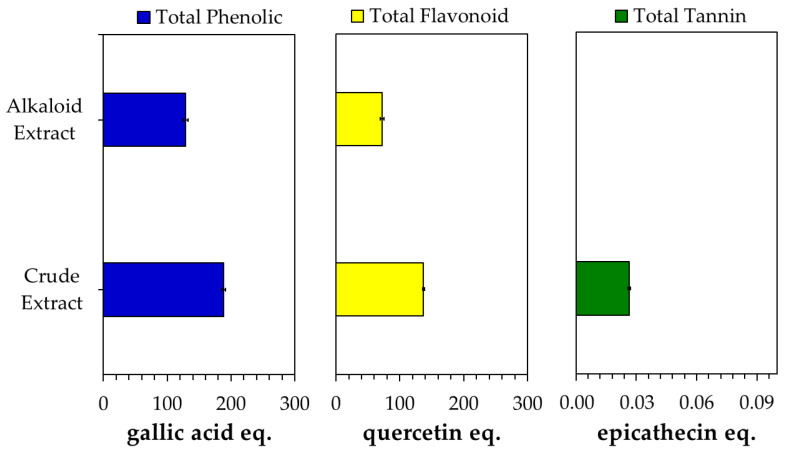
Phytochemical content of crude and alkaloid extracts obtained from Kratom.

An acid–base treatment was applied, followed by the continuation of the extraction process using non-polar solvents, e.g., hexane, chloroform/dichloromethane, to separate alkaloids, particularly MG, from non-alkaloid compounds via alkaloid salt/basic formation. In the acid condition, Kratom’s alkaloids such as MG form a salt, which makes them soluble in the acidic aqueous solution. This condition allows the alkaloids to be easily separated from non-polar compounds such as lipids by washing the solution with non-polar solvents like hexane. After being separated, the basification with an ammonia solution led the alkaloid salts to reform into their original compounds in a water phase, which could be easily removed by chlorogenic solvents like chloroform or dichloromethane. The separation of non-alkaloid components could be easily indicated by the change in the color of the alkaloid extract to a lighter color than the crude extract (Figure S4, Supplementary Information). Moreover, the alkaloid extract solution exhibited clearer signals of the HPLC chromatogram than the crude extract, with the peak enhancements of some alkaloids like MG, PAY and SPG (Figure S3, Supplementary Information). In addition, the phytochemical content of the alkaloid extract was found to be lower than that of the crude extract (Figure 3).

However, the MG content and other alkaloid compositions could vary depending on several factors, including the Kratom plant’s biology (variety, age), geographic growing site (climate, soil type) and harvesting period [45,46]. For instance, methanolic extraction in combination with the acid–base treatment and silica gel column chromatography separation successfully yielded 51–66% of MG (based on alkaloid extract) from Thai plant leaves, while about 12–25% of MG was obtained from Malaysian plant leaves [16,31,47,48]. In contrast, the predominant alkaloid in the plant grown at the University of Mississippi, USA, was mitraphylline (45%) instead of MG [45,49]. Furthermore, the selection extraction procedures significantly affect the selectivity and efficiency of MG extraction. McCurdy and co-workers obtained 34–39% of MG from ethanolic extraction combined with the acid–base treatment of Kratom leaves [39]. Applying ultrasonic irradiation (21.4 kHz, 70 W, 1 h, 25 °C) with methanol produced five times more MG than was obtained from common methanolic extraction (50 °C, 3 h) [50]. Ultrasonic irradiation with methanol was 1.3–1.7 more effective in extracting MG as well as 7-hydroxymitragynine (7-OHMG) compared with ethanol and acetonitrile [44]. 

The extraction was applied in pilot-scale facilities using a stainless-steel batch reactor with a stirrer (100 L) to obtain the performance information about the large-scale process. In general, upscaling the extraction did not significantly change the extract yield or MG productivity when the extraction process was conducted for 24 h in each step (Table 1, runs 1–2). However, shortening the extraction time to 3 h resulted in a significant reduction in the yield and MG productivity (Table 1, run 3). Here, we observed a significant formation of emulsion during the acid–base treatment in the alkaloid extraction step, which made the separation of the alkaloid phase difficult. HPLC analysis revealed that the emulsion contained a significant amount of MG (30–50%). As such, the separation in this emulsion phase should be strictly considered in order to minimize the loss of the products, particularly the alkaloid components.

### 2.3. Cytotoxic Activity of Kratom’s Extracts against A549 Cancer Cell Lines

**Figure 4:**
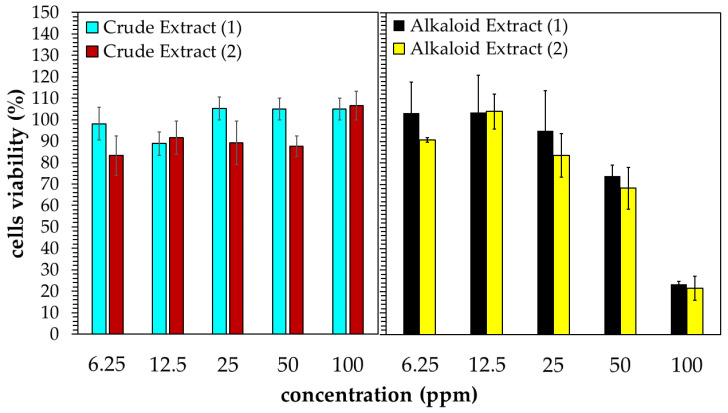
Cytotoxic activity of Kratom’s extracts obtained from laboratory- (1) and pilot-scale (2) experiments against A549 human lung cancer cells.

Figure 4 shows the in vitro test of the Kratom extracts’ cytotoxic activity against A549 human lung cancer cells after 24 h of incubation. The crude extract did not show any significant cytotoxic activity in the range of 6.25–100 ppm. An even greater proliferation of cancer cells was observed when the cells were exposed to the crude extract above 50 ppm. Contrarily, the alkaloid extract showed activity in a dose-dependent manner. The inhibition of cell proliferation was initially observed at the alkaloid concentration of 25 ppm. Moreover, about 80% of cancer cells could be inhibited at 100 ppm. It should be noted that the extracts obtained from the laboratory- and pilot-scale experiments yielded comparable results.

**Figure 5:**
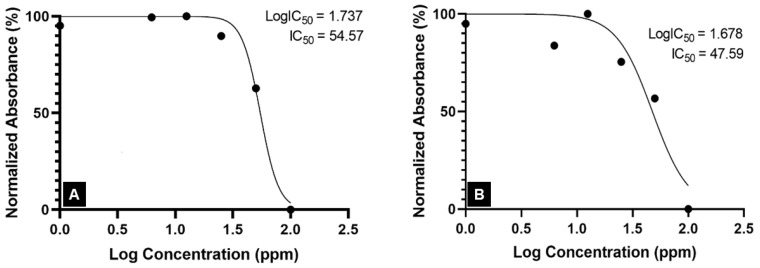
IC_50_ of Kratom alkaloid extract against the A549 lung cancer cell line: (**A**) lab-scale experiment and (**B**) pilot-scale experiment.

Alkaloids are the phytochemicals with the most promising anticancer properties since they can inhibit cancer cells by blocking the action of the topoisomerase enzyme, slowing DNA replication and promoting cell death [51]. The low MG content of the crude extract suggested low levels of alkaloid compounds in the extract, which might be responsible for the inactivity of the Kratom’s crude extract against A549 cancer cells. Meanwhile, both alkaloid extracts obtained from the laboratory- and pilot-scale experiments were rich in MG. Thus, they were active against A549 human lung cancer cells with IC_50_ values of 54.57 ppm and 47.59 ppm (Figure 5). Although these concentration ranges are associated with moderate cytotoxic activity [21], the results confirm the potential for alkaloids of Kratom to be used in cancer treatments. 

### 2.4. Cell Death Mechanism of A549 Cancer Cells toward Kratom’s Alkaloid

Considering the results concerning the cytotoxicity of alkaloids, further analysis is needed to investigate the effects of Kratom alkaloid treatment on the cell death mechanism against A549 human cancer cells. It is well known that cell death can occur via apoptosis and necrosis, which are morphologically and biochemically distinguishable. These morphological changes in cells can be seen using a fluorescence microscope with the Annexin V/propidium iodide (PI) staining protocol. Principally, phosphatidylserine is exposed to the outer surface of the plasma membrane in the early stage of apoptosis, and Annexin V works as an anticoagulant protein that binds to the phosphatidylserine with high affinity, resulting in fluorochrome-conjugated Annexin V that can be detected by a fluorescence microscope [52]. Therefore, this technique is good for determining apoptotic cells.

**Figure 6:**
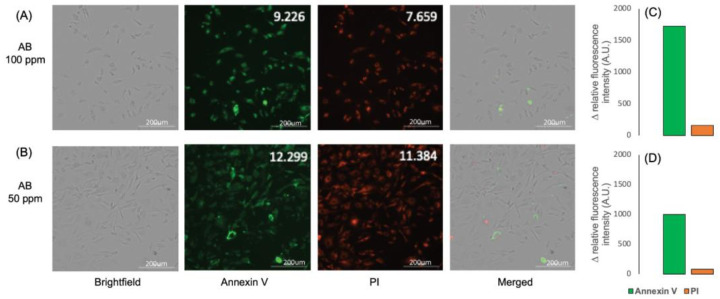
Imaging pictures of A549 cells treated with Kratom alkaloid extract at concentrations of (**A**) 100 and (**B**) 50 ppm. Annexin V and PI staining were used to determine the cell cycle. (**C**,**D**) show the relative fluorescent intensity difference between Annexin V and PI.

The A549 cancer cells were treated with the Kratom alkaloid extract at 50 and 100 ppm and were further analyzed using a fluorescence microscope with the Annexin V/PI staining protocol. The fluorescence imaging data indicate that A549 cancer cells treated with the Kratom alkaloid extract showed higher relative fluorescence intensity with Annexin than that in PI (Figure 6A,B). Taken together, a positive Annexin and a negative PI were exhibited, representing cells in early apoptosis (Figure 6C,D). It is known that apoptosis is a promising target for anticancer therapy. Apoptosis is the natural mechanism of cells for programmed cell death. Apoptosis plays a primary role in preventing cancer by controlling or possibly terminating the uncontrolled growth of cancer cells. Hence, targeting the cell’s mechanism of cell death is the most successful non-surgical cancer treatment [53]. These results indicate that the Kratom alkaloid extract is a promising substance for cancer therapy. 

### 2.5. Cytotoxic Activity of the Kratom Extract–Doxorubicin Combination against A549 Cancer Cell Lines

**Figure 7:**
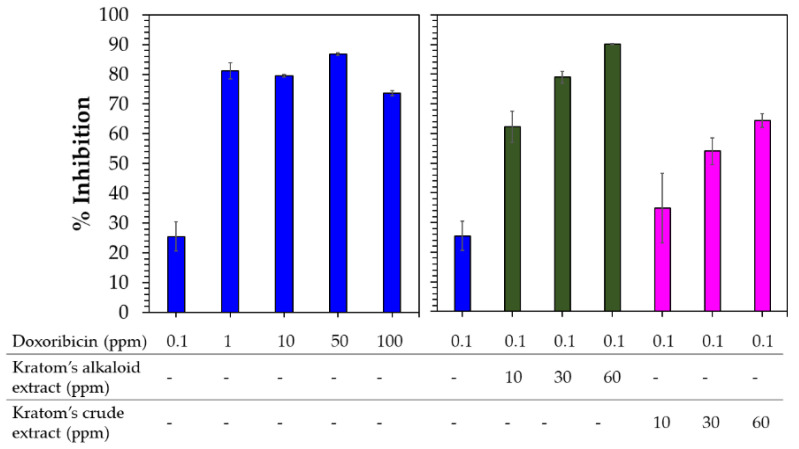
Cytotoxic activity of doxorubicin (**blue**) (**left**) compared with the combination of Kratom’s alkaloid extract (**green**) and Kratom’s crude extracts (**pink**) at several concentrations and 0.1 ppm doxorubicin (**right**) against A549 human lung cancer cells.

Domnic et al. reported that the use of alkaloid compounds of Kratom, i.e., MG and SPC, could lead to ~4- and >5-fold improvements in the cytotoxic activity of cisplatin against NPC/HK1 and C666-1 cancer cell lines, respectively [21]. Considering this result, we also investigated the effects of crude and alkaloid extracts of Kratom on the cytotoxic sensitivity of doxorubicin to A549 human cancer cell lines. Since doxorubicin showed potent anticancer activity against A549 human cancer cell lines (Figure 7), a dosage of 0.1 ppm of doxorubicin was selected to observe significant changes. Meanwhile, the extract was prepared at three concentrations—10, 30 and 60 ppm—which were selected based on the IC_50_ activity of the Kratom alkaloid extract, as discussed above (Figure 5).

Doxorubicin at a low dosage (0.1 ppm) showed 25% inhibition against A549 cancer cells (Figure 7). Above this concentration, doxorubicin significantly inhibited A549 cancer cells (70–90%). Combining the Kratom extracts with doxorubicin at concentrations higher than 0.1 ppm did not significantly affect the sensitivity of doxorubicin against A549 cancer cells due to its potent cytotoxic activity (Figure S5, Supplementary Information). As expected, the addition of the Kratom alkaloid extract enhanced the cytotoxicity of the low-dosage use of doxorubicin by 2.6 to 3.4 times. This combination is higher compared with the results of using the components alone. Interestingly, the use of a combination of the Kratom crude extracts also enhanced the sensitivity of doxorubicin by 1.3–2.4 times. In fact, the crude extract itself did not show any cytotoxic activity against A549 cancer cell lines (Figure 4). 

The greater sensitivity enhancement resulting from the combinatory usage of Kratom’s alkaloid extract compared to the crude extract could be related to its higher alkaloid components as represented by its MG content. According to a recent report, the combination of MG or SPC with cisplatin can improve the sensitivity of nasopharyngeal carcinoma cells to cisplatin by four times at a concentration close to its IC_50_ values compared with the use of cisplatin alone [21]. This activity was speculated to have occurred due to the ability of MG to inhibit mRNA expression and the protein expression of COX-2 induced by LPS [26]. In fact, COX-2 inhibitors can reduce inflammatory factors, thereby activating the antitumor immune microenvironment, downregulating vascular endothelial growth factor to inhibit tumor angiogenesis and inhibiting the PI3K/Akt signaling pathway to induce tumor cell apoptosis [54]. The overexpression of COX-2 is associated with tumorigenesis by stimulating proliferation, inhibiting apoptosis, promoting invasion by enhancing the production of matrix metalloproteinases and promoting angiogenesis [24,55]. In this respect, epidemiological studies and clinical trials have shown reductions in the incidence of various human cancers due to the long-term usage of COX-2 inhibitors such as celecoxib and rofecoxib [54]. For instance, the combined use of cisplatin and celecoxib was reported to enhance the sensitivity of nasopharyngeal carcinoma cells to cisplatin [25]. Therefore, a further study is being conducted in order to determine the ability of each alkaloid component in Kratom to regulate COX-2 expression in correlation with cytotoxic activities.

The calculated combination indices (CIs) for doxorubicin with the alkaloid and crude extract were 0.3 and 0.6, respectively. CI values below 1 indicate that the combinations of the alkaloid and crude extract with doxorubicin showed synergistic cytotoxic effects. These results provide a new potential alternative therapeutic for cancer treatment. The use of minimum concentrations with maximum activity in this combination strategy could significantly decrease the dose of doxorubicin used as a single agent in chemotherapy, which ranges from 60 to 75 mg/m^2^ intravenously and from 40 to 75 mg/m^2^ when administered in combination with other chemotherapy drugs. Hence, the side effects of doxorubicin as a sole drug, such as neuropathy, could be suppressed by combining it with the Kratom extracts which are known to have an opioid effect [56].

Since the crude extract contained a low MG content, which exhibited fewer alkaloids compared with the Kratom alkaloid extract, the presence of other metabolites might be responsible for the improvement in the effect of doxorubicin due to the addition of crude extract. Phytochemical analysis showed the significant presence of compounds other than alkaloids, such as phenolics, flavonoids and tannins. The latter were only detected in the crude extract. Since these phytochemicals could also affect cytotoxic activity [51], we assumed that the non-alkaloids in the Kratom extract could have a synergetic effect with doxorubicin. An intensive study is being conducted in our lab to obtain its mechanism of action.

## 3. Materials and Methods

### 3.1. Chemicals and Materials

Unless otherwise stated, all chemicals and reagents were of analytical-grade. Methanol, acetic acid, ammonium hydroxide, anhydrous sodium sulfate, hexane, chloroform and dichloromethane were purchased from Merck (Darmstadt, Germany). 

The Kratom leaf powder was obtained from local suppliers in Putussibau, Kapuas Hulu, West Borneo, Indonesia. It was derived from the red vein Kratom plants that grow frequently in this area. In general, the farmers harvested old leaves during the summer and retained four to six young leaves in a shoot. The harvested leaves were dried in the sun until the moisture of biomass was less than 10%. The dried leaves were then powdered by a local industry following a good manufacturing process, which separated the bone leaves and generated a green Kratom leaf powder. The sample was put in an airtight plastic bag and stored at room temperature for further analysis. According to the moisture analysis, the MG contents of the powder were 4.2% and 1.4%, with an average particle size of less than 50 μm (Figure S6, Supplementary Information).

### 3.2. Moisture Content Analysis

A sufficient amount of the Kratom leaf sample was vacuum-oven-dried at 105 °C for 24 h. Afterward, it was cooled down to room temperature and weighed. The moisture content was calculated as follows:

$$MC\left(\%\right)=\frac{W_{0}-W_{1}}{W_{0}}\times 100\%$$

where MC, W_0_ and W_1_ are the moisture content and the mass of the sample before and after drying, respectively.

### 3.3. Kratom Crude Extract Preparation

The Kratom leaf sample was extracted following the procedures used in previous studies [17,39]. Briefly, it was added to methanol (1:10 ratio) and stirred at room temperature for 24 h. Afterward, the mixture was filtrated by filter paper, and the spent biomass was re-extracted with fresh methanol under the same conditions. The extraction process was conducted until the filtrate solution became colorless (three-times repetition). Thereafter, the filtrates were collected and concentrated using a vacuum evaporator at 35 °C (Buchi Rotavapor R-300 Series, Buchi, Flawil, Switzerland). The obtained crude extract was then lyophilized (Buchi Lyovapor L-200, Buchi, Flawil, Switzerland) to obtain a dry crude extract. The yield was calculated from three replications using the equation below:

$$Yield\left(\%\right)=\frac{W_{DC}}{W_{s}-(W_{s}\times MC\%)}\times 100$$

where W_DC_ and W_S_ represent the mass of the dried crude extract and the mass of the sample, respectively.

### 3.4. Effect of Crude Extraction Conditions

Kratom leaf powder was extracted following the crude extraction protocol described above. The evaluation was based on the effects of three parameters—solvent selection, extraction time and temperature—on MG productivity in the obtained crude extract. The performances of several kinds of organic solvents, i.e., methanol, ethanol, 2-butanol, dichloromethane, chloroform and ethyl acetate, in the extraction process were evaluated. Meanwhile, extraction time was evaluated for 3–24 h using methanol as the best solvent. Furthermore, the extraction temperature was screened at 30–70 °C for 3 h under methanol. MG productivity was calculated by multiplying the obtained yield by the MG content of the extract.

### 3.5. Kratom Alkaloid Extract Preparation

An alkaloid extract was prepared as previously described [17,39]. In brief, the dried crude extract was dissolved in 10% acetic acid (1:6 ratios) and stirred well overnight at room temperature. Afterward, the liquid extract was filtered, and the solid residue was subjected to a fresh 10% acetic acid solution (in an equal amount to before) and stirred under the same conditions as before. The extraction process was conducted until a colorless liquid extract solution was obtained. Afterward, the filtrate solution was collected and washed with hexane (1:2 volume ratios) at least two times. The acid solution layer was collected and brought to a pH of 8–9 with 25% ammonia solution. The aqueous phase was then extracted with chloroform (1:2 ratio) six times. The chloroform layer was collected and washed with distilled water (1:1 ratio) two times. After separation, a sufficient amount of anhydrous sodium sulfate was added to the chloroform phase until the solution became clear. Thereafter, the remaining solids were separated using filtration, and the obtained chloroform solution was vacuum-dried at 35 °C. The yield was calculated from three replications using the equation below:

$${Yield}_{AL}\left(\%\right)=\frac{W_{AL}}{W_{DC}}\times 100$$

where W_AL_ and W_DC_ are the masses of the dried alkaloid and the dried crude extract. The yield of alkaloid from the original sample (Kratom leaves) could also be determined as follows:

$${Yield}_{AL}\left(\%\right)=\frac{{Yield}_{AL}\times W_{DC}}{W_{S}-(W_{S}\times MC\%)}\times 100$$

where W_s_ and MC% are the weight and moisture content (%) of the Kratom powder used.

### 3.6. Pilot-Scale Extraction

A stainless-steel stirrer reactor (100 L capacity) was used to perform pilot-scale extraction. The operation condition was adapted from the laboratory scale. 

### 3.7. Phytochemistry Tests

All the phytochemistry tests were performed using the same method used by Tang et al. (2020) [57]. The tests involved an extract with a concentration of 1000 ppm. The reaction was determined in a 96-well plate, and the absorbance was measured using a Tecan Spectrophotometer (Tecan, Männedrof, Switzerland).

#### 3.7.1. Determination of Total Phenolic Content (TPC)

The TPC value was determined using the Folin–Ciocalteu reagent by spectrophotometric method. In the 96-well plate, 25 µL extract was mixed with 25 µL Folin–Ciocalteu reagent solution (1:3 diluted with water) and 200 µL water. The mixture was incubated at room temperature for 5 min. Then, 25 µL 10% (*w*/*w*) sodium carbonate was added, and the mixture was incubated again for 60 min in a dark area. The absorbance was measured at 765 nm. TPC in samples was measured using a calibration curve with gallic acid as standard at concentrations ranging from 0 to 1000 µg/mL. The results were expressed as mg GAE per g dry weight (dw).

#### 3.7.2. Determination of Total Flavonoid Content (TFC)

The TFC value was determined using the aluminum chloride method. In a 96-well plate, 80 µL extract was mixed with 80 µL aluminum chloride (2% diluted in ethanol) and 120 µL of a 50 g/L sodium acetate solution. The mixture was incubated at 25 °C for 2.5 h. The absorbance was measured at 440 nm. The quercetin ranging from 0 to 50 µg/mL was used as the standard calibration curve. The TFC value was calculated as mg of quercetin equivalent per g (mg QE/g dw).

#### 3.7.3. Determination of Total Tannins Content (TTC)

The TTC value was determined using the p-dimethylaminocinnamaldehyde and vanillin methods. The extract of 25 µL was mixed with the same amount of 32% sulfuric acid and 150 µL of 4% vanillin solution (diluted in methanol). The mixture was incubated at 25 °C for 15 min, and the absorbance was measured at 500 nm. The TTC value was expressed as mg of epicatechin equivalent per g (mg CE/g dw). The calibration curve was prepared with catechin solution ranging from 0 to 1000 µg/mL.

### 3.8. HPLC Analysis

Crude and alkaloid extract solutions were prepared by adding a sufficient amount of extract to methanol. The sample solution was filtered through a 0.45 μm PTFE syringe filter and subjected to an HPLC system (UFLC Shimadzu, Kyoto, Japan) equipped with a photodiode array detector and Hypersil Gold C18 analytical column; 150 mm × 4.6 mm; 5 μm. The eluent used a mixture of acetonitrile (A) and water with TFA 0.03% (B), following the gradient elution program, as indicated in Table S2 (Supplementary Information), at a flow rate of 1.2 mL min^−1^. Chromatograms were monitored at a wavelength of 254 nm. Serial standard solutions with a linearity range of 1–100 ppm were prepared for quantification based on the peak area of the targeted compound. All solvents used liquid of chromatography grade. The typical chromatograms of sample extracts are displayed in Figure S1 (Supplementary Information).

### 3.9. Scanning Electron Microscopic Analysis

The morphology of the Kratom powder was observed using a scanning electron microscope (SEM Hitachi SU3500, Hitachi, Tokyo, Japan) at 1000× *g* magnification and 15.0 kV accelerating voltage. Before measurement, the sample was spread onto carbon tape paper and coated with Au using a 30 mA current for 10 s by ion sputter E-1045 (Hitachi Corp, Tokyo, Japan).

### 3.10. Cytotoxic Assays

The human lung cancer A549 cell line was purchased from the European Collection of Authenticated Cell Cultures (ECACC 86012804). The cells were cultured in a Dulbecco Modified Eagle Medium (DMEM) growth medium supplemented with 1% of antibiotic and antimycotic solution 100× (Sigma Aldrich, ST. Louis, MO, USA), 2 mM glutamine (Sigma Aldrich, ST. Louis, MO, USA) and 10% of fetal bovine serum (*v*/*v*) (Sigma Aldrich, ST. Louis, MO, USA). The cells were kept in a humidified incubator at 37 °C under 5% CO_2_ and passaged every two to three days. 

The cell viability was determined using an MTT (3-(4, 5-dimethylthiazolyl-2)-2, 5-diphenyltetrazolium bromide) assay according to Kumar et al. (2018) [58], with some modification. In brief, the cells were seeded in a 96-well plate at a density of 1 × 10^4^ cells/well and incubated at 37 °C for 24 h. All extracts for the in vitro assay were prepared using EtOH at a high concentration (100 mg/mL) and diluted using sterilized distilled water until 1 mg/mL. For the MTT assay, a diluted extract containing the medium was added to the wells to obtain a final concentration range of 6.25–100 μg mL^−1^ and a total volume of 200 μL well^−1^ (the final concentration of EtOH from the extracts in the well plate did not exceed 0.1%). The medium, doxorubicin and a blank medium were applied as a negative control, positive control and correction factor, respectively. After 24 h of incubation, a 100 μL medium was discarded, and about 10 μL of a 12 mm MTT (Sigma Aldrich, ST. Louis, MO, USA) solution in Dulbecco’s Phosphate Buffer Saline (Sigma Aldrich, ST. Louis, MO, USA) was added to each well. Thereafter, the mixture was incubated at 37 °C for 4 h. Finally, 150 μL of DMSO was added to each well and incubated at 37 °C for 10 min. The absorbance of each well was measured at 540 nm using Cytation 5, BioTek (Biotek, Winooski, VT, USA). The percentage of cell viability was calculated using the following equation:

$$Cell\;Viability\left(\%\right)=\frac{{(Abs}_{s}\times {Abs}_{b})}{{(Abs}_{c}-{Abs}_{b})}\times 100\%$$

where Abs_s_, Abs_b_ and Abs_c_ correspond to the absorbance of each well per extract, the absorbance of medium only without cells and the average absorbance of the cells without treatment, respectively. 

### 3.11. Cytotoxicity of Kratom–Doxorubicin Combination

The lung cancer A549 in 100 mL DMEM was seeded in each well in a 96-well plate and incubated at 37 °C under 5%CO_2_ for 24 h until the cells were well attached. The Kratom crude and alkaloid extracts were prepared using the same procedure as above (Point 3.4) and dispensed into each well in triplicate to give several final concentrations of 10, 30 and 60 ppm. The doxorubicin hydrochloride (Sigma-Aldrich) was prepared in the sterilized distilled water and added to cells cultured in a 96-well plate to give final concentrations of 0.1, 1, 10, 50 and 100 ppm. The combination assay was performed by combining aliquot doxorubicin with Kratom alkaloid extract and doxorubicin with Kratom crude extract to give a final doxorubicin concentration of 0.1 ppm and various concentrations of Kratom alkaloid extracts and Kratom crude extracts (10, 30 and 60 ppm). The cells were then incubated for 48 h at 37 °C under 5% CO_2_. After incubation, the media were discarded, 100 mL of 5% MTT solution was added and the media were incubated for an additional 4 h. After incubation, the MTT solution was removed, 100 mL DMSO was dispensed and the absorbance was measured at 570 nm using a microplate reader (Tecan, Männedrof, Switzerland).

### 3.12. Apoptosis Assays

The lung cancer A549 cells in 500 mL DMEM were seeded in each well in a 6-well plate and incubated at 37 °C under 5%CO_2_ for 24 h until the cells were well attached. Aliquots of EtOH 70% of Kratom alkaloid extract were dispensed into each well in triplicate to yield final concentrations of 50 and 100 ppm. The cells were then incubated for 24 h at 37 °C under 5% CO_2_. After incubation, the cells were treated with Annexin V and PI staining dye according to the kit’s protocol (ABP Bioscience-A025, Beltsville, MD, USA). The stained cells were then fixed in 4% paraformaldehyde and observed using BoTek Cytation 5 Cell Imaging (Agilent, Winooski, VT, USA).

## 4. Conclusions

The use of a single chemotherapeutic agent is still approved in the medical treatment of cancer. However, this technique often non-selectively targets actively proliferating cells, including healthy cells, and can make the cells resistant. Combination therapy offers several advantages over therapy using single chemotherapeutic agents, including lower toxicity to healthy cells, because different pathways are targeted, resulting in an improvement in treatment efficacy, the avoidance of drug resistance, and reduced treatment duration. Despite its attractiveness in activating human μ-opioid receptors, the alkaloids of Kratom (*Mitragyna speciosa* (Korth.) Havil. Rubiacease) have attracted much attention for their potential cytotoxic applications against several human cancer cell lines. Our in vitro study showed the sensitivity enhancement of doxorubicin at a low dosage (0.1 ppm) against A549 human cancer cell lines when it was combined with 10–60 ppm of the Kratom extract. The alkaloid extract resulted in 2.6- to 3.4-fold greater improvements than the crude extract addition since the former sole treatment inhibited cancer cells (IC_50_ of 48–55 ppm) via the apoptosis mechanism. Since the crude extract itself did not show any cytotoxicity against A549 cancer cell lines, the synergistic enhancement of the crude extract on doxorubicin suggests the potential contribution of non-alkaloids contained in the Kratom’s leaves to the observed activity. In general, the low (<1) calculated combination index (CI) of doxorubicin with Kratom’s extracts indicates that their potential synergistic combinations could reduce the dosage of doxorubicin used in chemotherapy.

## Figures and Tables

**Table 1:** Analysis results of leaf powder, crude and alkaloid extracts derived from Borneo Kratom using serial methanolic/acid–base extraction.

Sample		Run	Parameter	Unit	Values ^a^
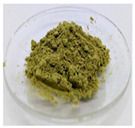	Kratom leaf powder		moisture content	%	4.18 ± 0.04
			MG content	%	~1.4 ^b^
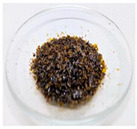	Kratom crude extract	1	crude extract yield ^c^	%	40.95 ± 0.70
			MG content	%	4.91 ± 0.14
		2	crude extract yield ^c^	%	38.56 ± 3.48
			MG content	%	4.50
		3	crude extract yield ^c^	%	25.00
			MG content	%	5.03
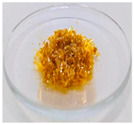	Kratom alkaloid extract	1	alkaloid extract yield ^c^	%	3.27 ± 0.11
			MG content	%	45.86 ± 1.61
		2	alkaloid extract yield ^c^	%	2.05
			MG content	%	43.6
		3	alkaloid extract yield ^c^	%	1.70
			MG content	%	38.56

Run 1 = laboratory scale, room temperature, 24 h extraction; run 2 = pilot scale (100 L), room temperature, 24 h extraction; run 3 = pilot scale (100 L), room temperature (3 h extraction); ^a^ calculated from three replications; ^b^ supplier information, ^c^ 24 h, room temperature; ^c^ based on the dry weight of leaf powder.

## Data Availability

Data are contained within the article and Supplementary Materials.

## Supplementary Materials

The following supporting information can be downloaded at https://www.mdpi.com/article/10.3390/molecules29061404/s1: Table S1. The color appearances and MG extraction; Table S2. Gradient program of eluent during HPLC analysis of Kratom extract; Figure S1. HPLC chromatograms of methanolic crude extract solutions obtained from different extraction solvents; Figure S2. UV absorbance of the MG peak in the alkaloid extract solution compared with the MG peak of the standard solution; Figure S3. Typical HPLC chromatogram of the crude (A) and MG-rich alkaloid extract. 7-OHMG, MG, PAY, SPG, SPC, A, B and C refer to 7-hydoxymitragynine, mitragynine, paynantheine, speciogynine, speciociliatine, kratom leaf powder, crude extract and alkaloid extract, respectively; Figure S4. Physical changes in color appearances for each step during serial extraction of kratom leaf powder using methanolic and acid–base treatments; Figure S5. Cytotoxic activity of the combination of doxorubicin and the Kratom extracts at several concentrations against A549 human lung cancer cell lines; Figure S6. Physical appearance (A) and scanning electron microscopic image (B) of the Kratom leaf powder.
