# Supplementary material for: An In Vitro Examination of Whether Kratom Extracts Enhance the Cytotoxicity of Low-Dose Doxorubicin against A549 Human Lung Cancer Cells
Source: Molecules. 2024 Mar 21;29(6):1404. doi: 10.3390/molecules29061404 (PMC10974887; doi:10.3390/molecules29061404)
Supplement: Supplementary file 1 [file molecules-29-01404-s001.zip › molecules-2888346-supplementary.pdf]

SUPPLEMENTARY INFORMATION

An In Vitro Examination of Whether Kratom Extracts Enhance the Cytotoxicity of Low-Dose Doxorubicin against A549 Human Lung Cancer Cells

Asep Bayu [1,*], Siti Irma Rahmawati [1], Firmansyah Karim [1], Jonathan Ardhianto Panggabean [1], Dasilva Primarindu Nuswantari [1], Dwi Wahyu Indriani [1], Peni Ahmadi [1], Rendi Witular [2], Ni Luh Putu Indi Dharmayanti [3] and Masteria Yunovilsa Putra [1,*]

[1] Research Center for Vaccine and Drugs, National Research and Innovation Agency (BRIN), Jalan Raya Jakarta-Bogor KM 46, Cibinong, Bogor 16911, Indonesia; siti.irma.rahmawati@brin.go.id (S.I.R.); firmansyahkarim93@gmail.com (F.K.); jonathanardhianto@gmail.com (J.A.P.); dasilvarindu@gmail.com (D.P.N.); dwiw007@brin.go.id (D.W.I.); peni.ahmadi@brin.go.id (P.A.)

[2] Ministry of Health, Republic of Indonesia, Jalan HR Rasuna Said Blok X5 Kavling 4-9, Kuningan, South Jakarta 12950, Indonesia; rendyaw@yahoo.com

[3] Research Organization for Health, National Research and Innovation Agency (BRIN), Jalan Raya Jakarta-Bogor KM. 46, Cibinong, Bogor 16911, Indonesia; nilu011@brin.go.id

* Correspondence: asep044@brin.go.id (A.B.); masteria.yunovilsa.putra@brin.go.id (M.Y.P.)

Table S1. The color appearances and MG extraction

Run	MG relative extraction (%)	Extract solution color	
		1st run	5th run
2	88	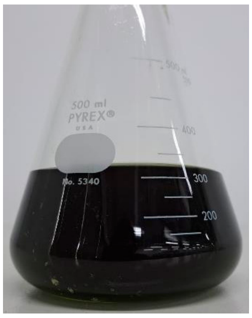	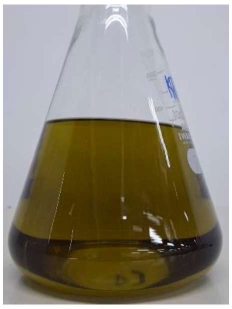
3	74		
4	16		
5	n.d		

Table S2. Gradient program of eluent during HPLC analysis of Kratom's extracts

**Time (min)**	**Mobile Phase A (%)**	**Mobile Phase B (%)**
0.01 – 4.00	70 → 30	30 → 70
4.01 – 6.00	30	70
6.01 – 8.00	30 → 70	70 → 30
8.01 – 20.00	70	30

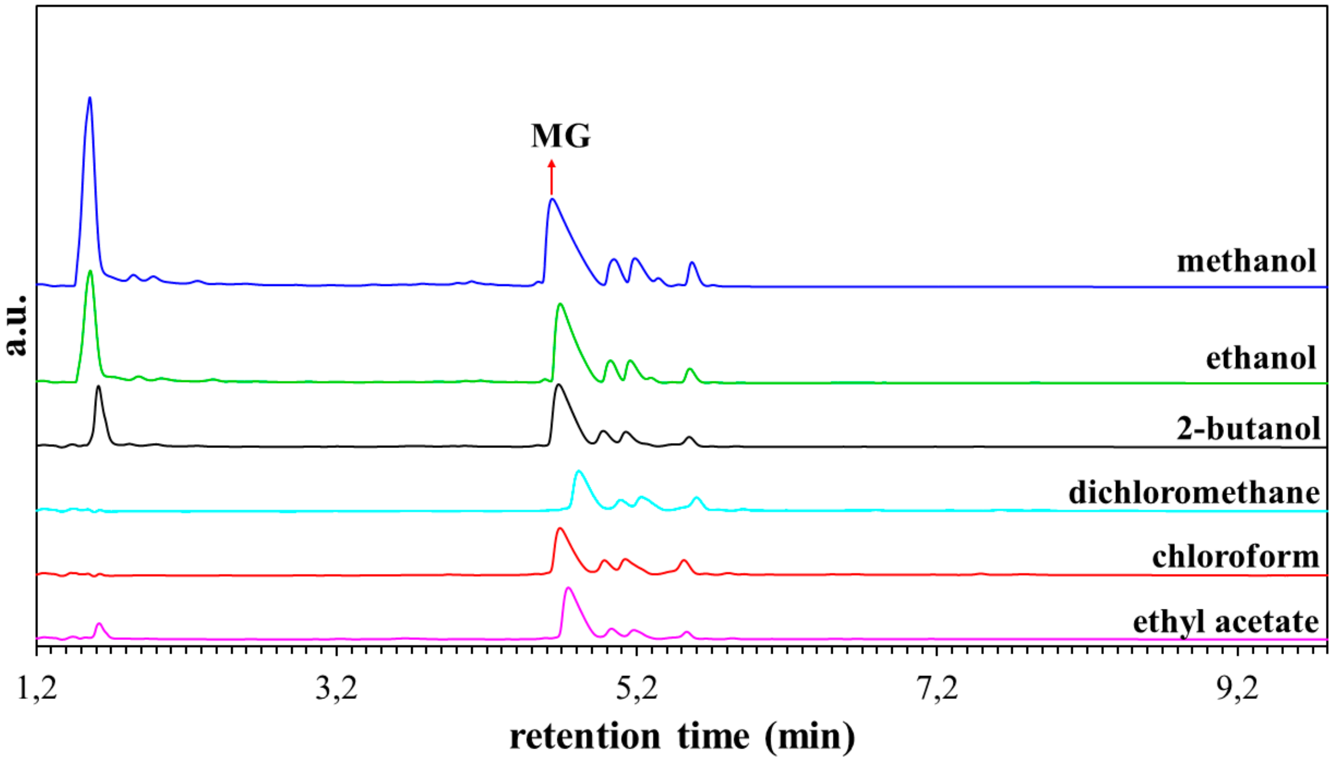

Figure S1. HPLC chromatograms of methanolic crude extracts solution obtained from different extraction solvents.

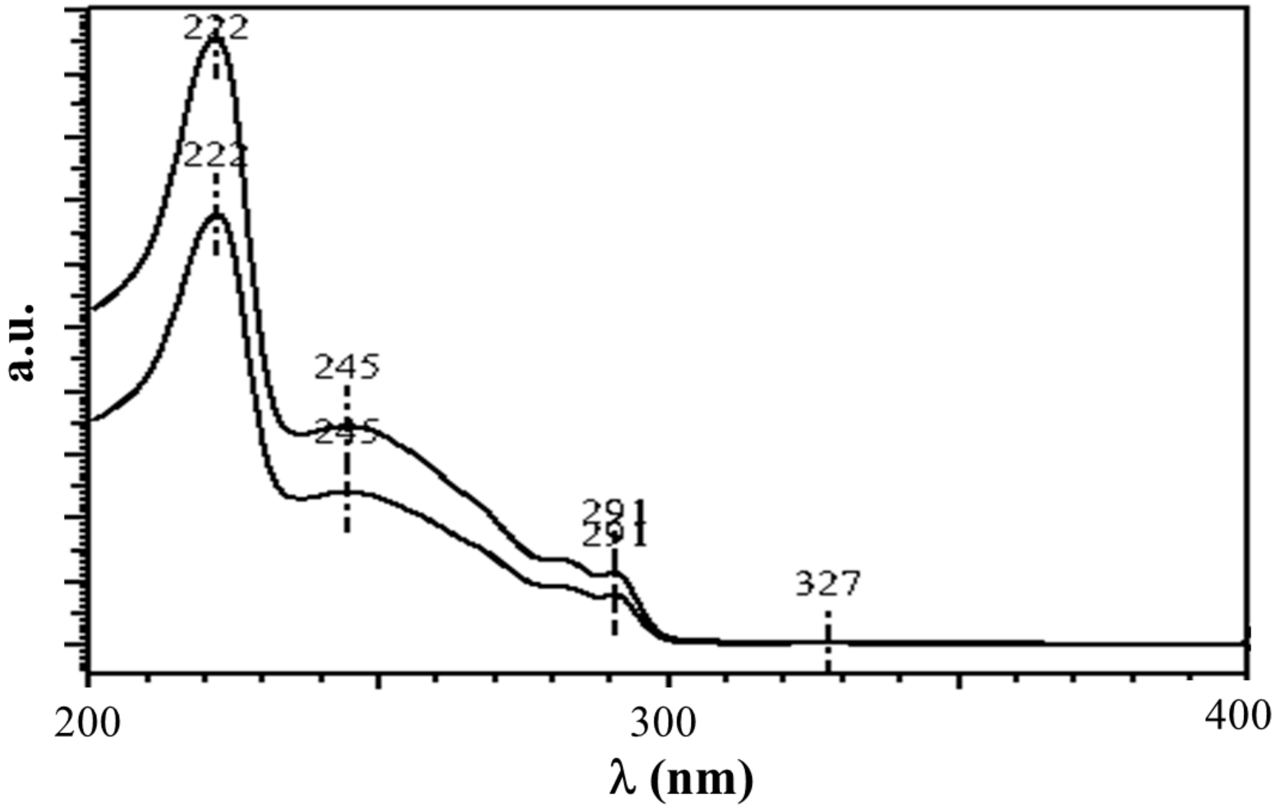

Figure S2. UV absorbance of MG peak in the alkaloid extract solution compared with MG peak of the standard solution.

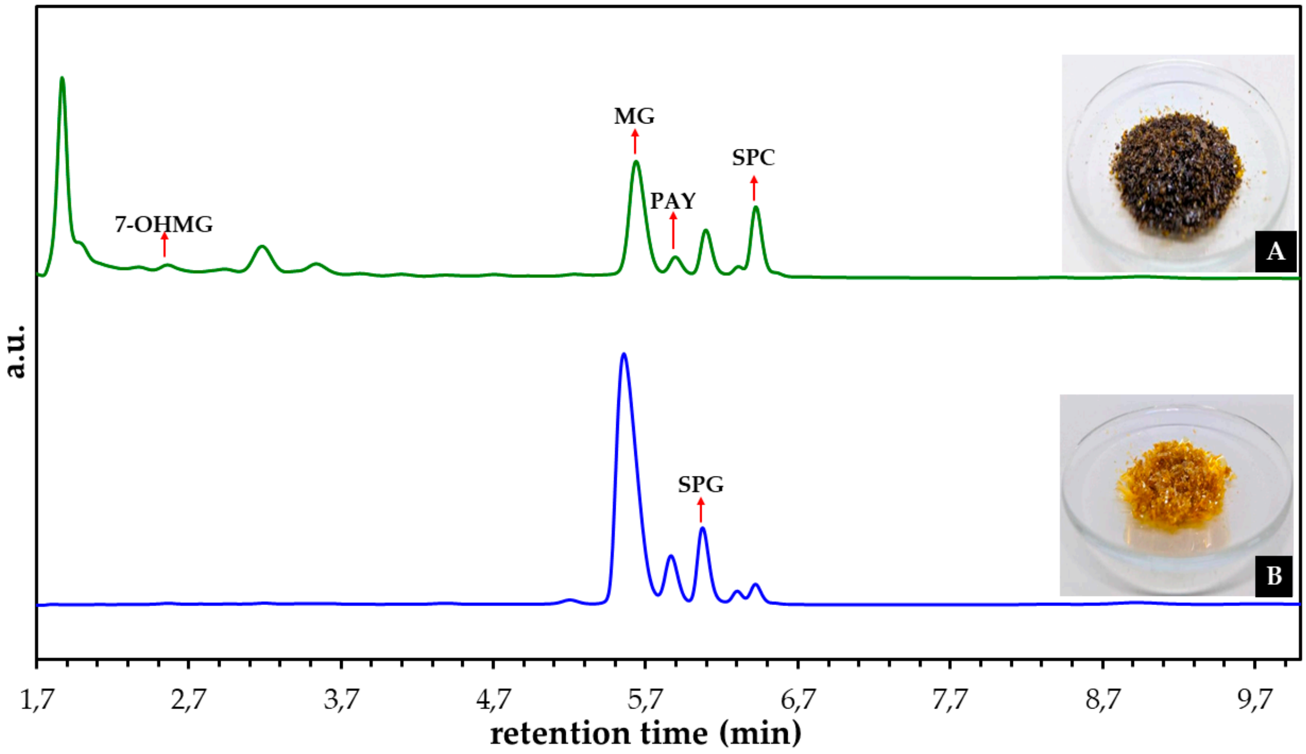

Figure S3. Typical HPLC chromatogram of the crude (A) and MG-rich alkaloid extract. 7-OHMG, MG, PAY, SPG, SPC, A, B, C refer to 7-hydoxymitragynine, mitragynine, paynantheine, speciogynine, speciociliatine, kratom leaves powder, crude extract and alkaloid extract, respectively.

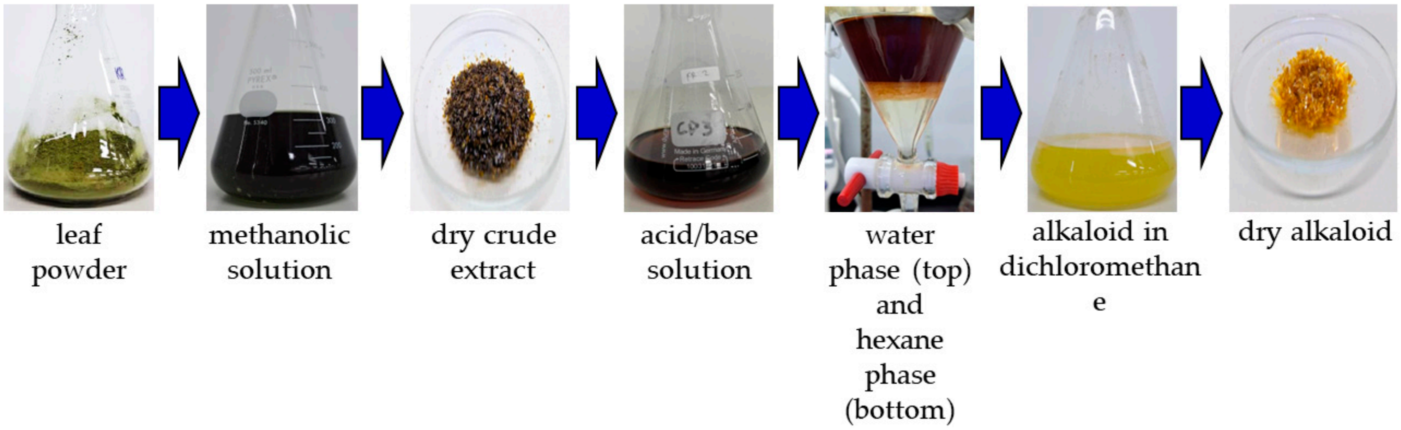

Figure S4. Physical changes of color appearances for each steps during serial extraction of kratom leaves powder using methanolic and acid/base treatments

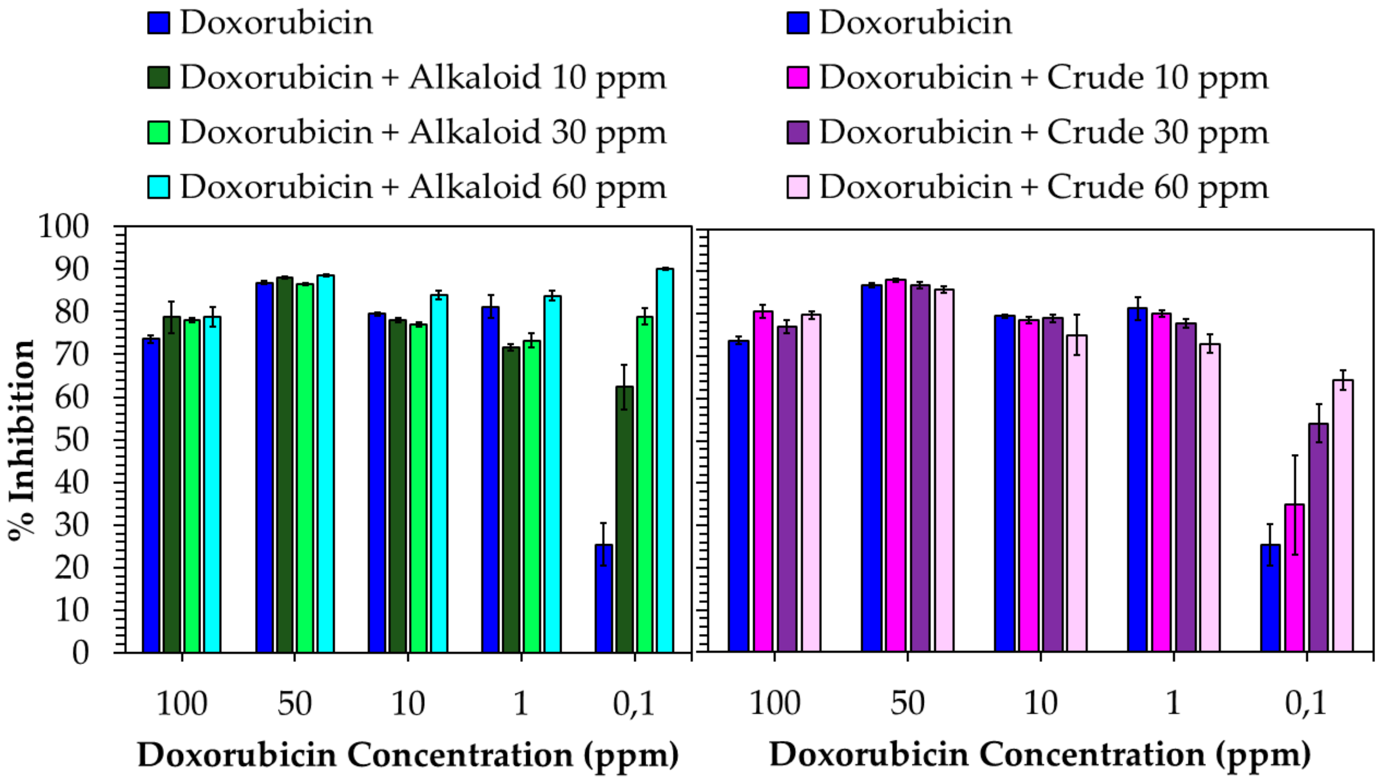

Figure S5. Cytotoxic activity of combination of doxorubicin and the Kratom extracts at several concentration against A549 human lung cancer cell lines.

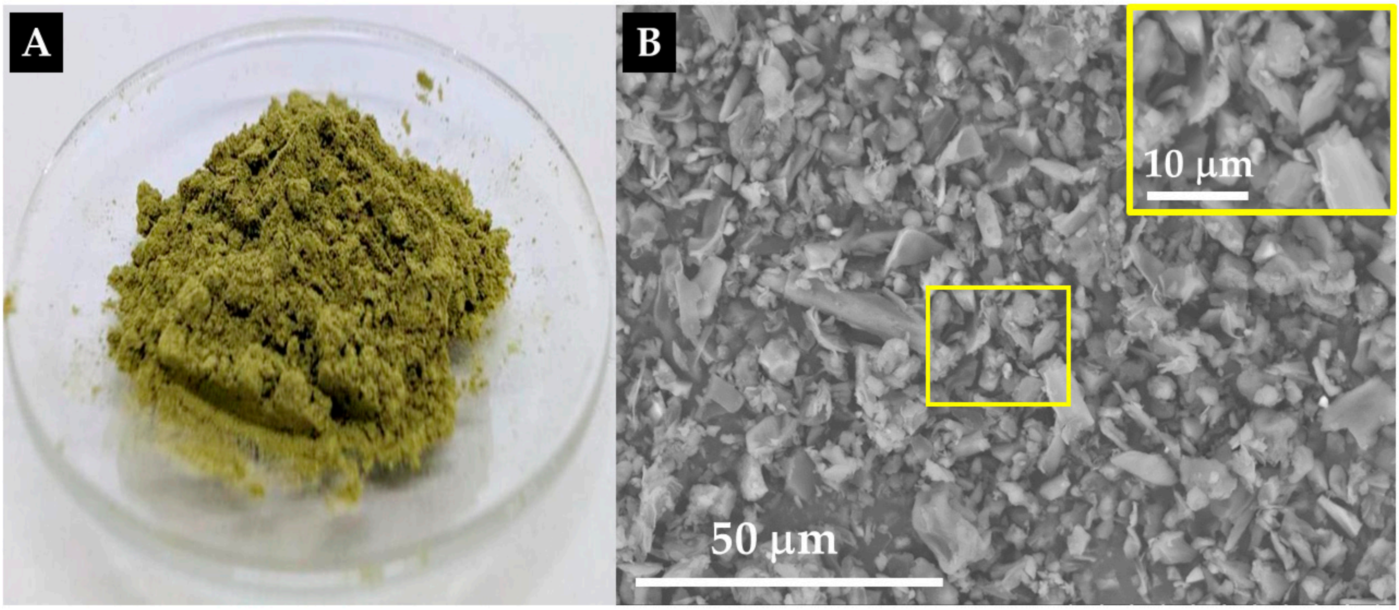

Figure S6. Physical appearances (A) and scanning electron microscopic image (B) of the kratom leaves powder.
